# Giant Mesenteric Cyst in a Young Adult Mimicking Refractory Ascites: A Diagnostic and Surgical Challenge—A Case Report

**DOI:** 10.1155/crgm/7405161

**Published:** 2025-04-23

**Authors:** Abate Bane Shewaye, Kaleb Assefa Berhane, Amanuel Getu Gebresilassie, Amsalework Daniel Fanta, Megersa Regassa, Fekadu Ayalew, Eyerusalem Fekede, Biruk Demisse Ayalew

**Affiliations:** ^1^Department of Internal Medicine, College of Health Sciences, Addis Ababa University, Addis Ababa, Ethiopia; ^2^Department of Internal Medicine, Adera Medical and Surgical Center, Addis Ababa, Ethiopia; ^3^Department of Surgery, Adera Medical and Surgical Center, Addis Ababa, Ethiopia; ^4^Department of Radiology, Adera Medical and Surgical Center, Addis Ababa, Ethiopia; ^5^Department of Pathology, ONCO Pathology Diagnostic Center, Addis Ababa, Ethiopia; ^6^Department of Internal Medicine, Saint Paul's Hospital Millennium Medical College, Addis Ababa, Ethiopia

**Keywords:** abdominal distention, case report, diagnostic and therapeutic challenge, Ethiopia, giant mesenteric cyst

## Abstract

Mesenteric cysts are rare benign intra-abdominal tumors that are usually asymptomatic and diagnosed incidentally while being investigated for other conditions or their complications. Surgical excision remains the primary treatment option. Here we present the case of a 30-year-old male with progressive abdominal distension, initially misdiagnosed with liver disease and refractory ascites, leading to inappropriate diuretic therapy. Subsequent imaging revealed a giant mesenteric cyst, which was successfully managed with complete surgical excision.

## 1. Introduction

Mesenteric cysts are benign cystic lesions that can occur in any part of the mesentery of the gastrointestinal (GI) tract, ranging from the duodenum to the rectum. They most commonly originate from the mesentery of the small bowel, followed by the mesocolon [1–3].

The exact cause of mesenteric cysts remains uncertain, though several theories have been proposed. A widely accepted theory suggests that mesenteric cysts arise from the benign proliferation of ectopic lymphatics in the mesentery, which lack proper communication with the rest of the lymphatic system [4]. Other potential factors include a failure of lymph nodes to connect with the lymphatic or venous systems, blockage of the lymphatic system due to trauma, infection, or neoplasms, and complications from previous pelvic surgery, pelvic inflammatory disease, or endometriosis [5, 6].

Mesenteric cysts are often asymptomatic, with approximately 40% of cases being discovered incidentally during physical examinations or investigations for other conditions. When symptoms do occur, they are variable and nonspecific. Common symptoms include abdominal pain (82%), nausea and vomiting (45%), constipation (27%), and diarrhea (6%). A palpable abdominal mass may be detected in more than 50% of patients during physical examination [1, 3, 6]. Additionally, mesenteric cysts can present as an acute abdomen due to complications such as intestinal obstruction, volvulus, torsion, bleeding, rupture, infection, or intestinal ischemia [1–3, 7].

Diagnosis is typically made using imaging techniques such as ultrasound, computed tomography (CT), or magnetic resonance imaging (MRI), with confirmation through histological examination [8, 9]. A CT scan is crucial for localizing the cystic mass and assessing the involved anatomical structures. Complete surgical excision is the preferred treatment to prevent recurrence and possible malignant transformation, which occurs in about 3% of cases [1, 2, 8].

Here, we present a rare case of a giant mesenteric cyst, managed surgically, in a 30-year-old male who initially presented with abdominal distention misdiagnosed as refractory ascites, emphasizing the diagnostic challenges and the critical role of imaging in early recognition.

## 2. Case Report

A 30-year-old male farmer from West Shewa in the Oromia region of Ethiopia presented to our outpatient department with a three-year history of progressive, painless abdominal distension, accompanied by intermittent vomiting of ingested food and indigestion. He had no history of changes in bowel habits, hematemesis, melena, or urinary symptoms. His personal and family medical history and surgical history were unremarkable.

He visited several medical centers prior to his presentation, undergoing multiple ultrasound examinations that were reported as ascites. He was misdiagnosed with liver disease and treated with diuretics and had multiple therapeutic peritoneal taps. Nevertheless, his abdominal distention persisted and was labeled as refractory ascites.

**Figure 1:**
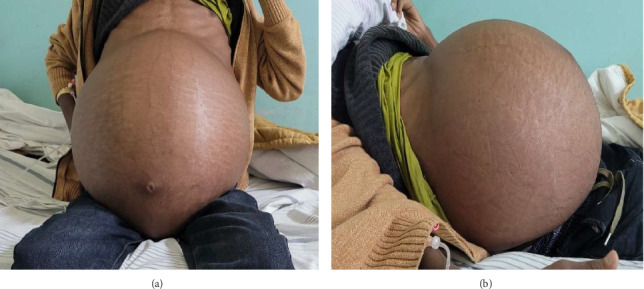
(a, b) Distended abdomen at the time of presentation.

Upon presentation to our medical center, the patient appeared chronically ill and slim, but had a body mass index (BMI) of 26.23 (weight 85.4 kg, height 180 cm) with stable vital signs and a grossly distended, nontender abdomen with abdominal collaterals (Figures 1(a) and 1(b)). The other system examinations were unremarkable.

Laboratory investigations revealed a reactive C-reactive protein (CRP), a mildly elevated erythrocyte sedimentation rate (ESR) of 25 mm/hr, and +2 leukocytosis on urinalysis. Complete blood count (CBC), liver and renal function tests, and serum albumin levels were within normal limits. Rapid diagnostic tests for hepatitis B surface antigen (HBsAg), hepatitis C virus antibodies (HCVAb), and human immunodeficiency virus (HIV) were negative. The tumor markers, carcinoembryonic antigen (CEA), cancer antigen 19-9 (CA 19-9), and cancer antigen 125 (CA 125), were all within normal limits.

**Figure 2:**
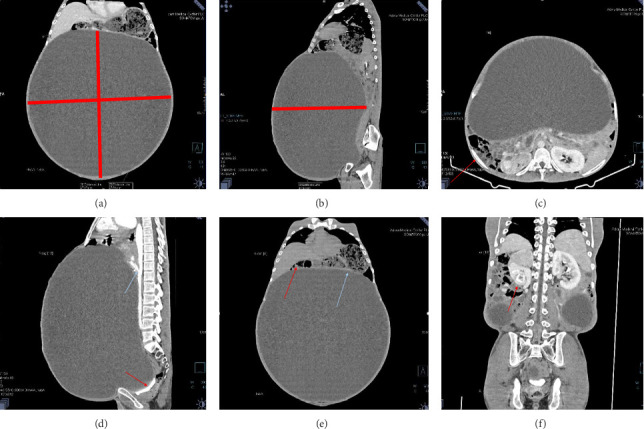
Contrast-enhanced CT of the abdomen showing a well-defined, large cystic lesion measuring 43.1 × 43.0 × 31.26 cm. (a, b) Axial and sagittal views of the cyst, (c) compression and displacement of intestinal loops, (d) compression of major vessels (blue arrow) and urinary bladder (red arrow), (e) displacement of the liver (red arrow) and spleen (blue arrow), (f) right-sided severe hydronephrosis.

Contrast-enhanced computed tomography (CECT) of the abdomen revealed a well-defined 43.1 × 43.0 × 31.26 cm abdominopelvic contrast nonenhancing cystic mass containing homogenous fluid higher than water attenuation without visible septation, soft tissue component, or calcification displacing bowel loops posterolaterally, the liver and spleen superiorly, and the urinary bladder inferiorly. The mass also compressed the right ureter, inferior vena cava (IVC), and extrahepatic portal vein with visible retroperitoneal collaterals. The hepatic veins were patent and well-opacified. There was marked right pelvicalyceal and ipsilateral proximal ureteric dilatation (Table 1 and Figure 2). There was no peritoneal thickening or mesenteric fat stranding.

With the diagnosis of a huge abdominopelvic cystic mass, likely a mesenteric cyst with mass effect and right severe hydronephrosis, exploratory laparotomy through a midline incision was performed after informed consent on his second day of presentation to our center.

**Figure 3:**
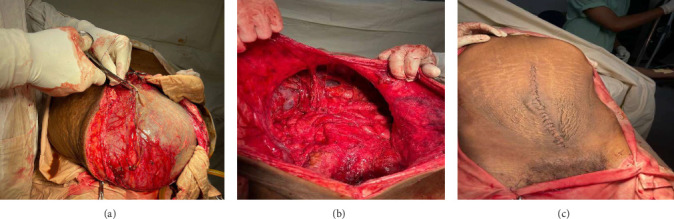
(a) Intraoperative image showing a massive, unruptured mesenteric cyst with a distended, translucent wall and prominent vascularization. (b) Post-excision image revealing the abdominal cavity after complete cyst removal. (c) Postoperative image of the abdomen after a well-approximated midline incision with intact sutures.

**Figure 4:**
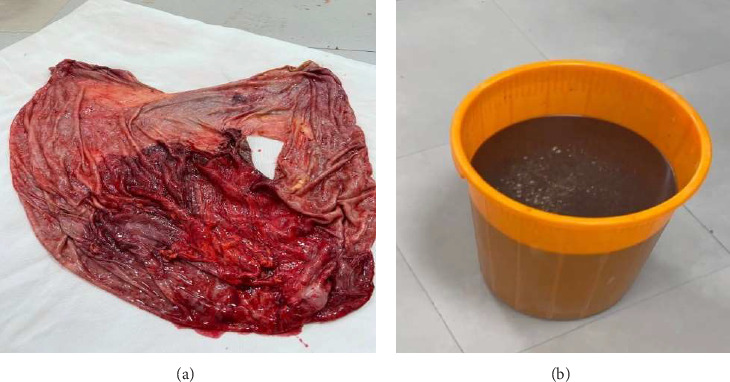
(a) Image of unilocular ruptured cystic tissue with wall thickness of 0.3 cm. (b) Dark brown fluid drained out of the unilocular cyst.

Intraoperatively, a large intra-abdominal cystic mass was identified, originating from the small bowel mesenteric base and compressing the entire small and large intestines posteriorly, as well as major abdominal vessels, the duodenum, stomach, and right ureter. The mass had soft adhesions to the small bowel, ureter, and retroperitoneal structures, which were carefully dissected and released without difficulty. It was approached with gentle right- and left-sided mobilization to minimize trauma to surrounding retroperitoneal organs and abdominal vessels. However, due to its large size and fluid content, intraoperative rupture occurred. This was managed by clamping the cystic sac with artery forceps and cautiously mobilizing the mass to prevent spillage and ensure safe removal. The cyst was successfully excised, with controlled intraoperative spillage (Figures 3(a) and 3(b)). Approximately 35 L of dark brown fluid was evacuated, and the cystic sac was sent for further histopathological analysis (Figures 3(a), 3(b), and 4).

**Figure 5:**
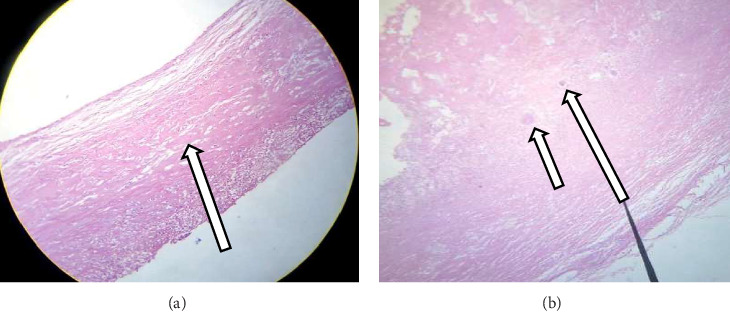
(a) Fibrotic cyst wall with no lining (40x). (b) Multinucleate giant cells.

The histological analysis of the excised cystic mass revealed cyst wall with no epithelial lining having epitheloid cell aggregates, multinucleate giant cells, and inflammatory cells favoring a benign mesenteric pseudocyst (Figure 5). Analysis of the cystic fluid reported an elevated protein content of 4.8 g/dL and high lactate dehydrogenase (LDH) levels at 600 U/L, suggesting an inflammatory process. Glucose levels were within the normal range at 76 mg/dL. Cholesterol was measured at 154 mg/dL, triglycerides at 90 mg/dL, and the pH level was 7.3. Cytological examination showed a predominance of inflammatory cells, including 28 cells/μL of neutrophils, 25 cells/μL of macrophages (elevated), and 21 cells/μL of lymphocytes, with some necrotic tissue present. Both Gram stain and acid-fast bacilli (AFB) tests were nonrevealing.

Postoperatively, the patient weighed 50.5 kg (BMI—16.2), and his abdominal girth significantly decreased (Figure 3(c)). Careful monitoring of fluid balance, electrolytes, and albumin was conducted. The patient was kept NPO for 24 h to manage potential ileus, with gradual reintroduction of meals once bowel function resumed. Complications, such as wound infection and seroma, were monitored, and early mobilization and respiratory support were encouraged to aid recovery. On his fourth day of admission, the patient was discharged home in stable and pleasant condition after a smooth postoperative course.

At the tenth-month postoperative follow-up visit, the patient remained asymptomatic, with normal clinical examination, abdominal sonographic findings, and laboratory results.

## 3. Discussion

Mesenteric cyst was first described by Benevenni, an Italian anatomist, in 1507 after performing an autopsy of an 8-year-old boy, while the first successful surgery for a mesenteric cystic mass was performed by Tillaux in 1803 [1, 10].

Mesenteric cysts are rare conditions, with fewer than 1000 cases reported in the literature so far. These cysts can develop at any age, with an incidence of approximately 1 in 100,000–250,000 adult hospital admissions. While earlier studies suggested a higher prevalence in men, more recent research indicates a female predominance, with a female-to-male ratio of 2:1 [3, 11]. However, our case involves a male patient. Mesenteric cysts are most commonly found in the small bowel mesentery (60%, particularly the ileum), followed by the large bowel mesentery (24%, mainly the ascending colon), and the retroperitoneum (14.5%), with 1.5% of cases having an indefinite site [9, 12]. In our patient, the mesenteric cyst originated from the proximal third of the jejunal mesentery.

Mesenteric cysts can be simple or multiple, uni or multiloculated, and they may contain hemorrhagic, serous, chylous, or infected fluid [1, 13]. The most often used classification was developed by de Perrot et al. in 2000, which divided mesenteric cysts into six groups based on histological characteristics: mature cyst teratoma, lymphatic cyst, urogenital cyst, mesothelial cyst, enteric cyst, and pseudocyst [14–16]. As shown in our case, the cyst walls of mesenteric pseudocysts are composed of fibrous tissue and lack an epithelial lining. These cysts typically develop as a result of trauma or infection and are believed to be a hematoma in the mesentery or omentum, or a residual abscess that has not fully resolved. Consequently, the internal contents of the cyst often consist of hemorrhagic material or purulent substances [17].

Mesenteric cysts can vary in size, ranging from a few millimeters to several centimeters in diameter. The increase in size is slow, progressive, and noticed late, and in about 18%–20% of cases, they grow so large that they resemble ascites, like in our case [3, 5, 13]. Similarly, a case reported in 2023 involved a 29-year-old female from Pakistan who presented with a seven-year history of abdominal distension resembling ascites. Abdominal CECT revealed a massive mesenteric cyst mimicking ascites, and surgical exploration confirmed the diagnosis, with histopathology ruling out malignancy [18]. Large mesenteric cysts can compress major abdominal vessels, compromising intestinal blood flow and increasing the risk of ischemia. Additionally, large cysts may displace the bowel, causing obstruction, volvulus, or intussusception further compounding the risk of ischemic complications [19]. In our case, despite the cyst's size, no ischemic signs were observed, likely due to gradual enlargement allowing vascular adaptation. However, prolonged compression could lead to venous congestion or infarction. The largest mesenteric cyst previously reported in the literature measured 30.4 × 31.7 × 24.0 cm on abdominal CT, with a gross weight of 16 kg, in a 46-year-old female from Cameroon who presented with a 9-month history of progressive abdominal distension [20]. In comparison, our patient's cyst measured approximately 43.1 × 43.0 × 31.26 cm and grossly weighed around 35 kg.

Diagnosis of mesenteric cysts can be challenging, as cysts mimic other pathologies, such as pancreatic pseudocysts or cystic tumors, pelvic diseases, and aortic aneurysms [1, 2]. Ovarian cysts are located in the adnexa and may show internal septations or solid components, while pancreatic pseudocysts are near the pancreas with a history of pancreatitis and signs of previous inflammation. These features may help differentiate mesenteric cysts from other conditions. A case involving an 8-year-old female who presented with abdominal pain, nausea, vomiting, and fever revealed a 10.5 × 8.7 × 7 cm cystic mass on CT, initially suspected to be ovarian in origin. However, diagnostic laparoscopy identified the cyst extending to the root of the mesentery. The mass was completely resected via laparotomy, and histopathological analysis confirmed it to be a benign mesenteric cyst [21].

In developing countries with high tuberculosis endemicity, mesenteric cysts can mimic tubercular ascites. Tuberculous ascites presents as free abdominal fluid without a well-defined cystic mass, often with peritoneal thickening or mesenteric fat stranding. Dulger et al. reported a case of a 25-year-old female whose mesenteric cyst was initially misdiagnosed as tubercular ascites, with no improvement observed following antituberculosis treatment. Upon further investigation, a CT scan was performed, which revealed a giant mesenteric cyst, which was then surgically removed [22].

A preoperative diagnosis can be made using imaging techniques (ultrasonography, CT, and nuclear MRI) [8, 9]. Abdominal ultrasound can be useful in the initial evaluation of an abdominal mass and can show a well-defined, fluid-filled cystic structure adjacent to bowel loops and fluid levels due to chyle and lymph. Abdominal CT scan is essential to localize the cystic mass and demonstrate the relationship between the cyst and nearby structures and blood vessels; it also helps in adequately planning the surgical approach [6, 8]. Diagnosis is then proven on laparotomy and has to be histologically confirmed [1], as lesions have been found to be cystic lymphangiomas, cystic stromal tumors, and mesotheliomas on a pathologic analysis [8]. Our patient had undergone multiple ultrasound examinations at other institutions all of which were interpreted as ascites. However, a CT scan was not performed due to availability constraint.

Mesenteric cysts and cystic lymphangiomas share similar clinical and radiological features but differ histologically. Small lymphoid aggregates in the cyst wall help distinguish lymphangiomas, which are rarer and more common in childhood. While typically benign, lymphangiomas can sometimes invade surrounding tissues, making accurate diagnosis essential [11].

Secondary complications associated with mesenteric cysts include volvulus, rupture, hemorrhage, torsion, spillage of infective fluid, herniation of the bowel into an abdominal defect, and obstruction [1, 3]. A case report was published of a 50-year-old male patient with a huge mesenteric cyst misdiagnosed clinically as obstructed inguinal hernia for 18 months who later presented with acute abdomen with a CT scan revealing a giant mesenteric cyst herniating into the right inguinal canal with gastric perforation [23].

In low-resource settings, where advanced imaging may be limited, clinicians should consider mesenteric cysts in the differential diagnosis of abdominal distention, especially when ascites does not respond to treatment. Early recognition can be aided by clinical examination and basic imaging like ultrasonography, which may reveal a well-defined fluid-filled mass. While CT and MRI are ideal for confirmation, improving awareness of this rare condition and providing access to basic imaging can enhance early diagnosis and timely intervention. Collaboration with referral centers for advanced imaging, when needed, can further improve outcomes.

A complete surgical excision is the first-choice therapy to avoid recurrence and possible malignant transformation, which may require the removal of part of the mesentery with the cystic mass. It can be done either by laparotomy or laparoscopy; the decision on the approach depends on the size of the cyst and its relationship with major abdominal structures, as well as the surgeon's experience [2, 8]. Partial excision is associated with recurrence and morbidity, and simple drainage should not be performed due to the associated high mortality and recurrence rate [8, 24]. Laparoscopic excision has been successfully performed for mesenteric cysts, even in large cysts, offering benefits such as reduced postoperative pain, shorter hospital stays, and faster recovery. Alenazi et al. reported a successful laparoscopic excision of a 10 × 10 × 9 cm mesenteric pseudocyst in a 31-year-old male. Similarly, a case of a young female with a large mesenteric cyst, measuring 27 cm in craniocaudal diameter, was successfully resected laparoscopically [25, 26]. However, in our case, given the cyst's enormous size and its extensive mass effect on surrounding structures, a laparoscopic approach was deemed technically challenging and unfeasible. Thus, open laparotomy was chosen as the preferred surgical option to ensure complete excision and minimize the risk of intraoperative complications.

Mesenteric cysts typically have a favorable prognosis due to their benign nature and low recurrence rates after complete excision. Due to the rarity of mesenteric cysts, data on malignancy risk are limited. Malignant transformation is rare, with Kurtz et al. reporting a 3% incidence, primarily low-grade sarcomas in adults. Recurrence rates range from 0% to 13.6%, with an average of 6.1% in a series of 162 adults and children. Recurrences are more common in patients with retroperitoneal cysts or those who underwent partial excision. Follow-up periods reported in the literature range from 3 to 48 months with ultrasound imaging, but long-term monitoring offers limited benefit as recurrences are rare and typically occur early [11, 24, 27]. In our case, no recurrence was observed after 10 months. Similarly, a case involving a calcified mesenteric cyst in a 70-year-old Ethiopian female showed no recurrence one year after complete surgical excision [11]. Given the benign nature of mesenteric pseudocysts, recurrence is rare following complete surgical excision. Our patient was advised to undergo periodic clinical and radiological assessments, with abdominal ultrasound after a year. Signs of recurrence, such as progressive abdominal distension, discomfort, or unexplained GI symptoms, should prompt further evaluation.

## 4. Conclusion

This case highlights the importance of considering mesenteric cysts in patients with unexplained abdominal distension and suspected ascites, particularly in settings where advanced imaging is limited. Early recognition and complete surgical excision are key to optimal patient outcomes.

## Figures and Tables

**Table 1:** Summary of CECT findings of the mesenteric cyst.

Size	Contents	Compression effects
43.1 × 43.0 × 31.26 cm	Homogenous fluid higher than water attenuation	Bowel loops, liver, spleen, urinary bladder inferiorly, right ureter, inferior vena cava (IVC), and extrahepatic portal vein

## Data Availability

The data that support the findings of this study are available from the corresponding author upon reasonable request.

## Nomenclature

AFB: Acid-fast bacilli
BMI: Body mass index
CBC: Complete blood count
CEA: Carcinoembryonic antigen
CECT: Contrast-enhanced computed tomography
CRP: C-reactive protein
CT: Computed tomography
ESR: Erythrocyte sedimentation rate
GI: Gastrointestinal
HBsAg: Hepatitis B surface antigen
HCVAb: Hepatitis C virus antibodies
HIV: Human immunodeficiency virus
IVC: Inferior vena cava
LDH: Lactate dehydrogenase
MRI: Magnetic resonance imaging
